# IP_3_R attenuates oxidative stress and inflammation damage in smoking‐induced COPD by promoting autophagy

**DOI:** 10.1111/jcmm.16546

**Published:** 2021-05-31

**Authors:** Qiang Zhang, Wei Li, Nahemuguli Ayidaerhan, Wuxin Han, Yingying Chen, Wei Song, Yuanyi Yue

**Affiliations:** ^1^ Department of Pulmonary and Critical Care Medicine Shengjing Hospital of China Medical University Shenyang China; ^2^ Key Laboratory of Intelligent Computing in Medical Image Ministry of Education Northeastern University Shenyang China; ^3^ Department of Pulmonary and Critical Care Medicine Tarbagatay Prefecture People’s Hospital Tacheng China; ^4^ Department of Clinical Laboratory Tarbagatay Prefecture People’s Hospital Tacheng China; ^5^ Department of Gastroenterology Medicine Shengjing Hospital of China Medical University Shenyang China

**Keywords:** autophagy, inflammation, IP_3_R, obstructive pulmonary disease, oxidative stress

## Abstract

Tobacco smoking is one of the most important risk factors for chronic obstructive pulmonary disease (COPD). However, the most critical genes and proteins remain poorly understood. Therefore, we aimed to investigate these hub genes and proteins in tobacco smoke‐induced COPD, together with the potential mechanism(s). Differentially expressed genes (DEGs) were analysed between smokers and patients with COPD. mRNA expression and protein expression of IP_3_R were confirmed in patients with COPD and extracted smoke solution (ESS)‐treated human bronchial epithelial (HBE) cells. Moreover, expression of oxidative stress, inflammatory cytokines and/or autophagy‐related protein was tested when IP_3_R was silenced or overexpressed in ESS‐treated and/or 3‐MA‐treated cells. A total of 30 DEGs were obtained between patients with COPD and smoker samples. IP_3_R was identified as one of the key targets in tobacco smoke‐induced COPD. In addition, IP_3_R was significantly decreased in patients with COPD and ESS‐treated cells. Loss of IP_3_R statistically increased expression of oxidative stress and inflammatory cytokines in ESS‐treated HBE cells, and overexpression of IP_3_R reversed the above functions. Furthermore, the autophagy‐related proteins (Atg5, LC3 and Beclin1) were statistically decreased, and p62 was increased by silencing of IP_3_R cells, while overexpression of IP_3_R showed contrary results. Additionally, we detected that administration of 3‐MA significantly reversed the protective effects of IP_3_R overexpression on ESS‐induced oxidative stress and inflammatory injury. Our results suggest that IP_3_R might exert a protective role against ESS‐induced oxidative stress and inflammation damage in HBE cells. These protective effects might be associated with promoting autophagy.

## 1 INTRODUCTION

Chronic obstructive pulmonary disease (COPD), a general term for a series of airflow limitation diseases, has been predicted to become the third leading cause of death in 2030 by the World Health Organization (WHO). In recent years, COPD seriously threatened the health of humans and reduced the quality of normal life. The symptoms of patients with COPD always include chronic cough, sputum production and dyspnoea, which are mainly associated with the inflammatory response in the airway and alveolar epithelium.^1^ Symptoms could be induced by a variety of reasons, among them, tobacco smoking, especially in elderly smokers, is one of the main risk factors for COPD.^2^ More than 4000 chemicals are contained in tobacco smoke, of which at least 20 are carcinogenic substances.^3^ The pathogenic mechanisms of tobacco smoke mainly include inflammation, oxidative damage and cell senescence.^4^ In addition, previous studies have reported that changes in gene expression and epigenetic modification are related to tobacco smoke–induced inflammation;^1^, ^5^ meanwhile, a large number of protein‐coding genes are associated with COPD exacerbation.^6^, ^7^ However, the gene and protein whose alteration is most critical in tobacco smoke–induced COPD remain poorly understood. Therefore, first, this study used bioinformatics methods to explore the biomarkers of COPD induced by tobacco smoke based on the gene chip databases. We found inositol 1,4,5‐trisphosphate receptor was one of the common different expression genes of patients with COPD and normal smokers compared with the non‐smoker control, separately.

IP_3_R can regulate the release of intracellular Ca^2+^ through binding the second messenger IP3 from the endoplasmic reticulum (ER).^8^ In the molecule, IP_3_R is essentially regulated by IP3 and Ca^2+^,^9^ and intracellular signals such as the redox state,^10^ ATP^11^ and cAMP^12^ can modulate its regulation. Moreover, IP_3_R was found to play an important role in both normal physiological processes and disease. The central function of IP_3_R is its delivery of Ca^2+^ to mitochondria or lysosomes, by which it participates in regulatory processes such as oxidative phosphorylation and cell apoptosis.^13^, ^14^, ^15^ Dysregulation or mutation of IP_3_R is associated with neurodegenerative disorders.^16^, ^17^ In addition, IP_3_R signalling pathway might be a target for cancer therapeutics by inducing autophagy.^18^ The latest studies demonstrated that IP_3_R‐associated calcium signalling plays an essential role in the oxidative stress process in human endothelial cells.^19^ However, whether IP_3_R contributes to the smoke‐induced respiratory inflammatory response and oxidative stress remains not obvious.

Airway inflammation, which might be related to increased oxidative stress, is widespread in patients with tobacco smoke–induced COPD.^20^, ^21^ Numerous oxidants can be directly measured in tobacco smoke, and tobacco smoking can deplete antioxidants.^22^, ^23^ Moreover, oxidative stress remains high due to the presence of active inflammatory cells in tobacco smoke–induced COPD. The inflammatory response was also shown to be further amplified in this process by activation of the NF‐κB pathway.^24^ Multiple cell and proinflammatory cytokines participate in the inflammatory response.^25^ IL‐4, IL‐5, IL‐9 and IL‐13 mediate inflammation, whereas TNF‐α and IL‐1β further amplify the inflammatory response.^21^, ^26^ In tobacco smoke–induced COPD patients, an imbalance between oxidative and antioxidative processes caused by the inflammation response eventually leads to disease progression.^27^

Autophagy is a widespread basic activity in eukaryotic cells^28^ that plays an essential role in the inflammatory response to stress in the airway and lung.^29^, ^30^ Furthermore, there is increasing evidence that the autophagy process plays critical roles in the progression of COPD‐emphysema.^31^, ^32^, ^33^, ^34^, ^35^, ^36^ It has been demonstrated that exposure to tobacco and/or e‐cig/nicotine vapour promotes oxidative stress reaction and inflammatory response that could lead to autophagy‐flux impairment, further impeding the important cellular homeostatic processes involved in the removal of misfolded proteins and bacterial/viral pathogens, ultimately affecting cell survival.^33^, ^34^, ^37^ In this process, Ca^2+^ signalling is one of the basic mechanistic targets for modulating autophagic flux.^38^ Previous studies proposed that IP_3_R could mediate Ca^2+^ signalling as an essential target in starvation‐induced autophagy.^39^ Cytosolic Ca^2+^ buffering could impede autophagy through the mTOR‐related pathway.^40^ However, whether cell autophagy–related protein mediated by IP_3_R participates in the regulation of oxidative stress and inflammation induced by tobacco smoke requires further investigation.

In the present study, we performed a bioinformatics analysis to identify the potential molecular targets of tobacco smoke–induced COPD. A subsequent study was performed to verify the differential expression of one of these selected targets (IP_3_R). Finally, we aimed to explore the role of IP_3_R in the process of oxidative stress and inflammation in a tobacco smoke–induced human bronchial epithelial (HBE) cell injury model and determine whether IP_3_R regulation is associated with autophagy.

## 2 MATERIALS AND METHODS

### 2.1 Data resources

Gene expression data used in this study were obtained from the National Center for Biotechnology Information Gene Expression Omnibus (NCBI‐GEO) database. We obtained the gene expression data sets GSE54837 and GSE37768. Microarray data from these two data sets were collected with the GPL570 platform ([HG‐U133_Plus_2]). The GSE54837 data set comprised data from 136 COPD patient, 84 smoker control and six non‐smoker control samples. The GSE37768 data set comprised data from 18 COPD patient, 11 smoker control and nine non‐smoker control samples. The batch effects in the expression data were adjusted using distance‐weighted discrimination methods.^41^ Human blood samples were from Department of Pulmonary and Critical Care Medicine, Shengjing Hospital of China Medical University. The research ethics committee at Shengjing Hospital of China Medical University, China (ethics number: 2015PS173K), approved the study and its methodology. All study participants provided written informed consent. The study was conducted in accordance with the principles of the Declaration of Helsinki (2013).

### 2.2 Data pre‐processing of differentially expressed genes

Differentially expressed genes (DEGs) between COPD/non‐smoker controls and smoker/non‐smoker controls were identified via GE2O online tools. The DEGs between these pairs of groups were aggregated. A *P*‐value <.05 and a |logFC| > 2 were used as the cut‐off criteria for DEG screening. Then, raw data were analysed with the Venn online website to determine common DEGs between the COPD/non‐smoker control and smoker/non‐smoker pairs of groups.

### 2.3 Gene ontology and Kyoto Encyclopedia of Genes and Genomes (KEGG) pathway enrichment analyses

DAVID is an online bioinformatics tool that is widely used to identify gene and protein functions.^42^, ^43^ In the present study, gene ontology (GO) analysis of the enrichment of biological process (BP), molecular function (MF) and cellular component (CC) terms in the DEGs and KEGG pathway analysis were performed with the DAVID online tool.

### 2.4 Reagents

Human bronchial epithelial cells and lentiviruses were purchased from GeneChem. FBS, RPMI‐1640 medium and penicillin/streptomycin were obtained from HyClone. Commercial kits used for the analysis of CAT, MDA, SOD and GSH‐Px were purchased from Nanjing Jiancheng Bioengineering Institute. The 2′7′‐dichlorofluorescein diacetate (DCFH‐DA) used to measure reactive oxygen species (ROS) formation was obtained from Beyotime Biotechnology. A cell proliferation assay kit (MTS method) was obtained from Promega Biotech Company. TRIzol used for RNA extraction, a PrimeScript RT reagent kit, a SYBR Premix TaqTM kit and dT primers were all purchased from TaKaRa Biotechnology Company. Total protein extraction kits were purchased from Invent Biotechnologies Institution. A BCA reagent kit and SDS‐PAGE kit were obtained from Beyotime Biotechnology. PVDF membranes were purchased from Millipore. Antibodies against IP_3_R and fluorescently labelled secondary antibody were obtained from Cell Signaling Technology. Antibodies against Atg5, LC3, P62, Beclin1 and β‐actin were purchased from CST, and goat anti‐rabbit IgG HRP secondary antibody was obtained from the Absin Bioscience Institution. 3‐Methyladenine (3‐MA) was purchased from MedChemExpress.

### 2.5 HBE cell cultures and transfection

The HBE cell line was used in the present study. HBE cells were thawed and cultured in six‐well cell culture plates. RPMI‐1640 medium containing 10% FBS and 1% penicillin/streptomycin was used to culture the cells in an incubator set at 37°C and 5% CO_2_.

To explore the function of IP_3_R in tobacco smoke–induced oxidative stress and inflammation, we purchased lentiviruses expressing si‐RNA against IP_3_R, which were transduced into HBE cells according to the manufacturer’s instructions. In addition, HBE cells transduced with lentiviruses expressing control si‐RNA were used as a control group. Subsequently, IP_3_R was overexpressed in the experimental group with the same method, and HBE cells transfected with lentiviruses containing a scrambled sequence were used as the control group.

### 2.6 Pre‐treatment with aqueous cigarette smoke extract

Tobacco smoke was extracted based on previous reports.^44^, ^45^ Three cigarettes from different companies (Liqun, Nanjing, China; Hongtashan, Yunnan, China; and Jiaozi, Sichuan, China) were burned, and the smoke mixtures were extracted through a vacuum pump and dissolved in 10 mL of PBS. The pH of the extracted smoke solution (ESS) was adjusted to 7.4, and the ESS was filtered through a 0.22‐μm filter. HBE cells in the experimental group were cultured in medium containing 5% ESS. HBE cells cultured in medium containing a 5% extract of normal air prepared in a manner similar to that used to prepare the experimental extract were used as a control group.

### 2.7 IP3R double immunofluorescence of HBE cells

Human bronchial epithelial cells were pre‐treated for 1 hour with 5% ESS, and the medium was then replaced. After pre‐treatment for 24 hours, the HBE cells and medium were collected. HBE cells after ESS exposure and control group were fixed with formaldehyde, and treated with 0.1% Triton X‐100 to permeabilize the cell membrane. The cells were then blocked with non‐immunized animal serum and incubated with antibodies against IP3R (1:500; Cell Signaling Technology) at 4°C and incubated with a fluorescently labelled secondary antibody (1:200; Cell Signaling Technology) for 2 hours at room temperature. DAPI was applied to stain the nuclei, and results were imaged using a fluorescence microscope.

### 2.8 Determination of CAT, MDA, SOD and GSH‐Px levels

Human bronchial epithelial cells were pre‐treated for 1 hour with 5% ESS, and the medium was then replaced. After pre‐treatment for 24 hours, the HBE cells and medium were collected. The supernatant of the cell and medium mixture was recycled after sonication and centrifugation. Protein concentrations in the supernatant were determined with the BCA method. The absorbance of CAT, MDA, SOD and GSH‐Px at 405, 530, 450 and 412 nm, respectively, was determined and recorded according to the specifications of the assay kit manufacturer. The levels of MDA and the activities of CAT, SOD and GSH‐Px were calculated based on equations.

### 2.9 Measurement of ROS formation

Human bronchial epithelial cells were collected 24 hours after pre‐treatment for 1 hour with 5% ESS. Digested cells were harvested after centrifugation, and precipitated cells were resuspended in binding buffer after washing twice. The final concentration of HBE cells was 1 × 10^5^/mL. As shown in previous research,^46^ HBE cells were incubated with 10 μmol/L DCFH‐DA for 30 min in an incubator. Finally, ROS formation was quantified via flow cytometry (Becton Dickinson) and is shown as the fluorescence intensity.

### 2.10 Quantification of HBE cell viability

Human bronchial epithelial cell viability was quantified using the MTS method. HBE cells were evenly cultured in a 96‐well assay plate and pre‐treated for 1 hour with 5% ESS, following which the cell medium was recycled with fresh medium, and the incubation was continued for 24 hours. Subsequently, the cells were cultured in the incubator for 2 hours after the addition of 20 μL of MTS assay reagent to each well. Absorbance values at 490 nm were determined using a microplate reader (BioTek Epoch).

### 2.11 Real‐time quantitative PCR (RT‐qPCR) analysis

Human bronchial epithelial cells were collected after pre‐treatment with ESS as previously described. Gene expression levels of *TNF‐α*, *IL‐1β, IL‐4*, *IL‐6* and *IP_3_R* were determined via RT‐qPCR, and the primer sequences are provided in Table 1. *β‐Actin* was used as a housekeeping gene. Total RNA was isolated with TRIzol reagent, and cDNA was synthesized with a PrimeScript RT kit according to the manufacturer’s instructions. RT‐qPCR was performed with a SYBR Premix TaqTM Kit using a 7500 real‐time PCR system (Applied Biosystem). After pre‐denaturation for 30 seconds at 90^o^C, RT‐qPCR was performed as 40 cycles of denaturation for 5 seconds at 95°C and annealing for 34 seconds at 60°C. Relative target genes were quantified with the comparative CT ($2^{‐\Delta \Delta C_{t}}$) method and then compared with target gene expression in the control group. The gene expression of each target in each sample was measured in three independent experiments.

**TABLE 1:** Primer sequences used for RT‐qPCR in this study

Gene name	Gene ID		Primer sequences (5'‐3')
*IP_3_R*	NM_002222	Forward	GCGGAGGGATCGACAAATGG
		Reverse	TGGGACATAGCTTAAAGAGGCA
*TNF‐α*	NM_000594	Forward	CCTCTCTCTAATCAGCCCTCTG
		Reverse	GAGGACCTGGGAGTAGATGAG
*IL‐1β*	NM_000576	Forward	ATGATGGCTTATTACAGTGGCAA
		Reverse	GTCGGAGATTCGTAGCTGGA
*IL‐4*	NM_000589	Forward	CCAACTGCTTCCCCCTCTG
		Reverse	TCTGTTACGGTCAACTCGGTG
*IL‐6*	NM_000600	Forward	ACTCACCTCTTCAGAACGAATTG
		Reverse	CCATCTTTGGAAGGTTCAGGTTG
*β‐Actin*	NM_001101	Forward	CATGTACGTTGCTATCCAGGC
		Reverse	CTCCTTAATGTCACGCACGAT

### 2.12 Western blot

After pre‐treatment with ESS for 1 hour, HBE cells were collected 24 hour later. Total protein was extracted using an Invent protein extraction kit (containing 1% PMSF), and the protein concentration was quantified using BCA kits. Equal quantities of protein (30‐60 μg) were separated by 8%‐12% SDS‐PAGE and transferred to PVDF membranes. The membranes were blocked for 2 hours, followed by incubation with primary antibodies at 4°C overnight. The membranes were washed three times and incubated with HRP‐conjugated anti‐rabbit antibodies (1:5000) for 1 hour at room temperature. An enhanced ECL substrate was used to measure the optical densities of the bands. β‐Actin was used as the loading control. The relative optical densities of bands in the Western blots were determined with Fluor Chem 2.0. Representative protein bands from one single experiment are shown in the figures, and integrated density values indicating the relative expression of each protein are shown as a bar chart. Each protein western blot analysis was performed at least three times. Primary antibodies against the following were used in the present study: IP_3_R (1:800), Atg5 (1:1000), LC3 (1:1000), P62 (1:1000), Beclin1 (1:1000) and β‐actin (1:2500).

### 2.13 Statistical analysis

Statistical analyses in this study were performed with SPSS 17.0, and GraphPad Prism software was used to prepare figures. Student’s *t* test was used to analyse the variance between experimental groups and the control group. One‐way analysis of variance followed by the Student–Newman–Keuls (SNK) test was used for comparisons between multiple means. Sample data from independent experiments are shown as the mean ± standard deviation (SD). Differences for which *P < .05 or **P < .01 were considered statistically significant.

## 3 RESULTS

### 3.1 Determination of the DEGs common to smokers and COPD patients compared with non‐smoker control

**FIGURE 1:**
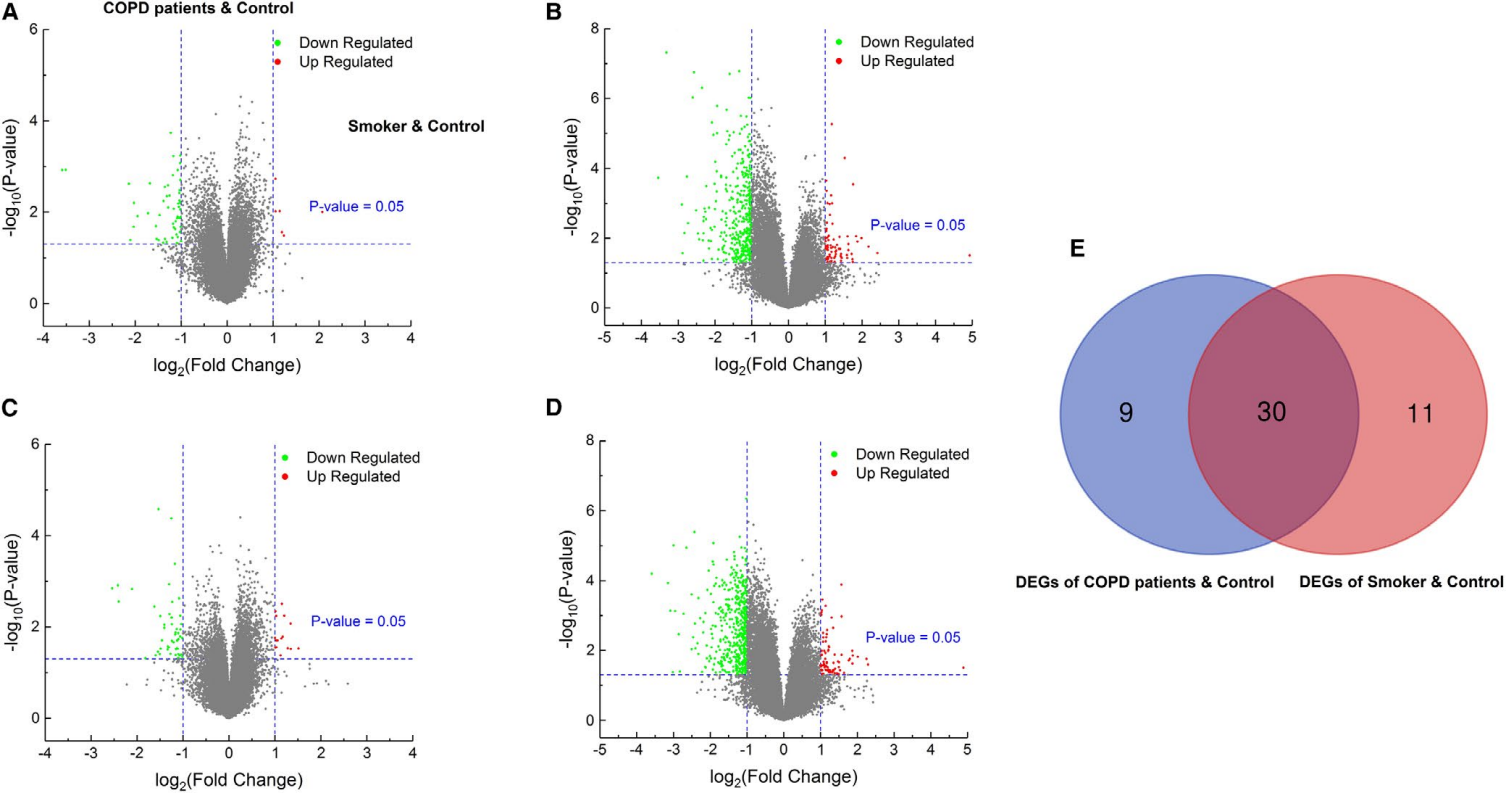
Determination of DEGs among COPD patients and smoker samples. A and B, Volcano plots of DEGs between COPD patient and non‐smoker control samples; C and D, Volcano plots of DEGs between smoker control and non‐smoker control samples; E, DEGs common to COPD patients and smoker samples via a Venn diagram

A total of 154 COPD patient samples, 95 smoker control samples and 15 non‐smoker control samples were used in the present study. From the GSE54837 and GSE37768 data sets, we obtained DEGs between COPD patient and non‐smoker control samples and DEGs between smoker and non‐smoker control samples via GEO2R. The results after preliminary screening are shown as volcano plots in Figure 1A‐D. Forty‐one DEGs between COPD patient samples and non‐smoker control samples were extracted, while 39 DEGs between smoker and non‐smoker control samples were extracted. Subsequently, we used the Venn diagram website to determine the DEGs common to smokers and COPD patients, and finally obtained 30 common DEGs (Table 2 and Figure 1E).

**TABLE 2:** All 30 commonly DEGs in COPD and smoker samples from GEO datasets

Gene name	DEGs	log FC	log FC
		(COPD patients & control)	(Smoker & control)
Calcium and integrin binding family member 4	CIB4	−3.54	−3.59
Guanylate cyclase 1 soluble subunit alpha 2	GUCY1A2	−2.51	−2.72
heparan sulfate 2‐O‐sulfotransferase 1	HS2ST1	−2.35	−2.43
Retinal G protein coupled receptor	RGR	−2.16	−2.13
Eukaryotic translation initiation factor 1A, Y‐linked	EIF1AY	−2.88	−2.83
Protocadherin related 15	PCDH15	−2.16	−2.01
HOXA distal transcript antisense RNA	HOTTIP	−2.57	−2.65
Phenylalanyl‐tRNA synthetase beta subunit	FARSB	−2.83	−3.08
Wilms tumor 1 associated protein	WTAP	−2.08	−2.04
Glucuronidase, beta pseudogene 4	GUSBP4	−2.73	−2.95
Centromere protein N	CENPN	−2.26	−2.19
Inositol 1,4,5‐Trisphosphate Receptor Type 1	ITPR1	−2.23	−2.18
Gap junction protein gamma 1	GJC1	−2.3	−2.13
X inactive specific transcript	XIST	4.93	4.89
Notch 3	NOTCH3	−3.32	−3
Zinc finger protein, Y‐linked	ZFY	−2.33	−2.51
Fibrillin 1	FBN1	−2.43	−2.32
Ubiquitously transcribed tetratricopeptide repeat containing, Y‐linked	UTY	−2.08	−2.4
Taxilin gamma pseudogene, Y‐linked	TXLNGY	−2.24	−2.42
Glycoprotein M6A	GPM6A	−2.11	−3.15
Phospholipase A2 group IIA	PLA2G2A	−2.03	−2.42
Long intergenic non‐protein coding RNA 491	LINC00491	−2.76	−2.47
MIR670 host gene	MIR670HG	−2.9	−2.86
Solute carrier family 24 member 3	SLC24A3	−2.6	−2.32
semaphorin 3A	SEMA3A	2.42	2.25
microRNA 675///H19, imprinted maternally expressed transcript	MIR675///H19	−2.13	−2.54
Uncharacterized LOC100288911	LOC100288911	−2.08	−2.35
SPANX family member A2///SPANX family member C///sperm protein associated with the nucleus, X‐linked, family member A1	SPANXA2///SPANXC///SPANXA1	−2.31	−2.09
Uncharacterized LOC100505902///uncharacterized LOC285389	LOC100505902///LOC285389	−2.46	−2.03
Long intergenic non‐protein coding RNA 1568	LINC01568	−2.28	−2.51

### 3.2 GO and KEGG pathway enrichment analyses of DEGs in smokers and COPD patients

The results of GO analysis and KEGG pathway related to the selected DEGs in tobacco smoke‐induced COPD are shown in Table 3 and Figure S1. For BP analysis, DEGs were particularly enriched in BP related to the process of visual perception. For MF analysis, DEGs were significantly enriched in MF related to calcium ion binding and ion channel activity. For CC analysis, DEGs were markedly enriched in CC related to the ER membrane and plasma membrane. For the KEGG pathway, the selected DEGs were significantly enriched in the vascular smooth muscle contraction process. In addition, the results showed that three genes, PLA2G2A, GUCY1A2 and IP_3_R, were enriched in this pathway. Based on the results of GO and KEGG pathway analyses, the *IP_3_R* gene was selected for subsequent experimental verification in the present study.

**TABLE 3:** GO and KEGG analyses of DEGs in tobacco smoke–induced COPD

Category	Term	Count	*P*‐value	Genes
GOTERM_MF_DIRECT	GO:0005509~calcium ion binding	7	.0001	NOTCH3, CIB4, SLC24A3, FBN1, PLA2G2A, PCDH15, ITPR1*
GOTERM_MF_DIRECT	GO:0005216~ion channel activity	2	.0449	ITPR1*, GJC1
GOTERM_CC_DIRECT	GO:0005789~endoplasmic reticulum membrane	4	.0373	NOTCH3, PLA2G2A, ITPR1*, GJC1
GOTERM_CC_DIRECT	GO:0005886~plasma membrane	8	.0493	NOTCH3, GPM6A, SLC24A3, PLA2G2A, GUCY1A2, PCDH15, ITPR1*, GJC1
GOTERM_BP_DIRECT	GO:0007601~visual perception	3	.0172	PCDH15, RGR, GJC1
KEGG_PATHWAY	hsa04270:Vascular smooth muscle contraction	3	.0075	PLA2G2A, GUCY1A2, ITPR1*

^*^ ITPR1 = IP_3_R.

### 3.3 IP_3_R was decreased in COPD and ESS‐treated HBE cells

**FIGURE 2:**
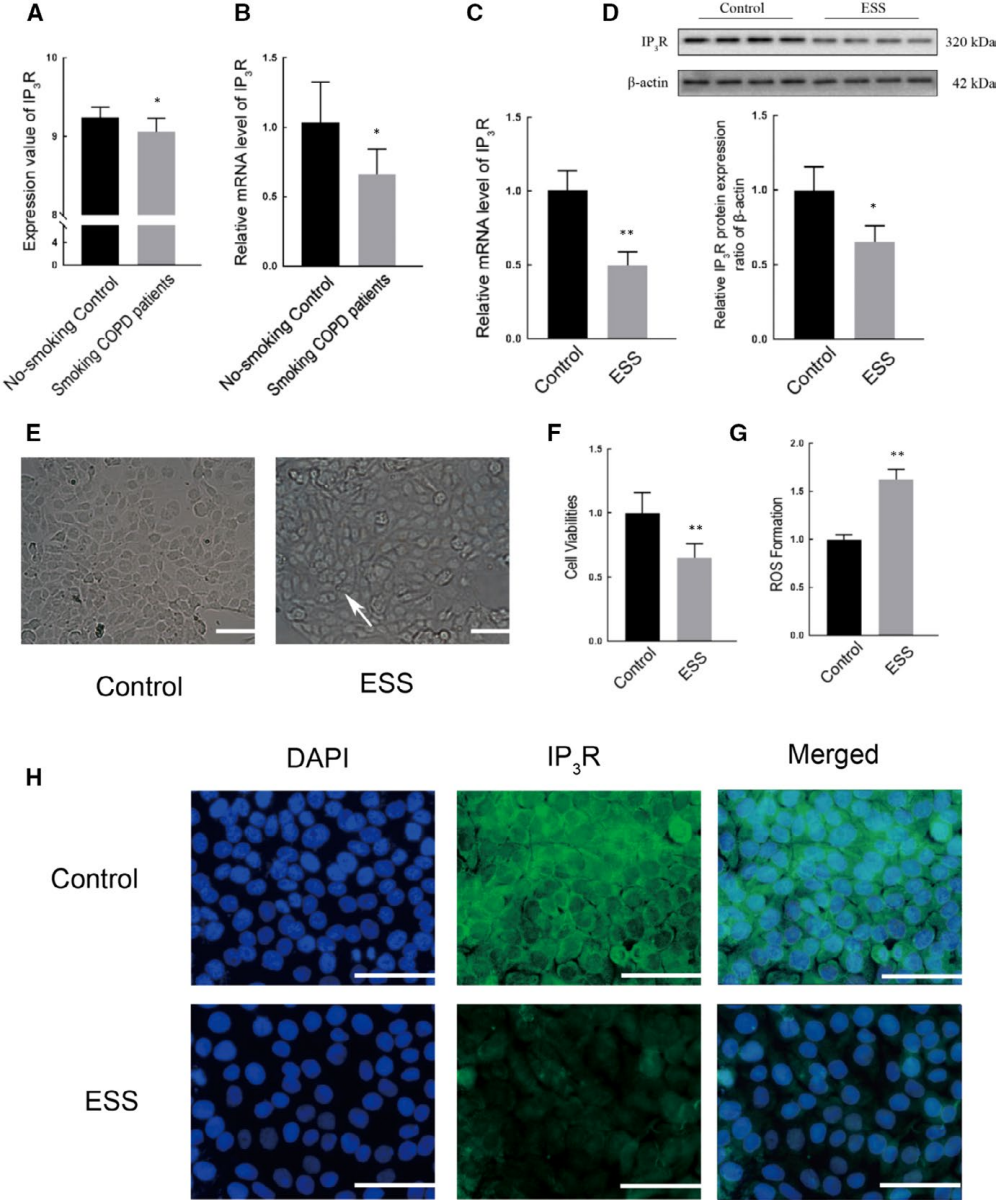
Verification of differences in IP_3_R expression and the effects of ESS on HBE cells. A, The analysis of IP_3_R mRNA expression data from smoking COPD patient and non‐smoker control samples in a GEO data set (GSE3365); B, the difference in IP_3_R gene expression in the peripheral blood of patients with COPD (who had smoked for more than 15 y) and volunteer non‐smokers (n = 6); C and D, the mRNA and protein expression of IP_3_R after treatment with ESS in HBE cells; E, morphological changes after treatment with ESS in HBE cells (200×); F, changes in HBE cell viability after exposure to ESS; G, ROS formation in HEB cells after exposure to ESS; H, immunofluorescence data of IP_3_R expression in HEB cells after exposure to ESS (400×). All experiments were performed at least three times, and similar results were obtained; the data are shown as the mean ± SD. *P < .05, **P <.01 compared with the control group

To verify the differential expression of the *IP_3_R* gene, we analysed its mRNA levels in tobacco smoke–induced COPD lung samples and non‐smoker control samples using a GEO data set (GES103174, 11739563_a_at). Figure 2A shows that the IP_3_R mRNA was significantly decreased in tobacco smoke–induced COPD patients compared with the controls (*P* < .05). In addition, we performed RT‐qPCR analysis to further determine the IP_3_R mRNA level in peripheral blood samples from patients with COPD (who had smoked for more than 15 years) and volunteer non‐smokers, which showed that the transcription of IP_3_R was also significantly reduced in the patients with COPD (*P* < .05, Figure 2B). Subsequently, we analysed both the mRNA expression and protein expression of IP_3_R in ESS‐treated HBE cells. The results in Figure 2C,D indicated that both the mRNA expression (*P* <.01) and protein expression (*P* <.05) of IP_3_R were markedly decreased after ESS exposure. In addition, microphotographs showed that the number of HBE cells was decreased and that the area of intercellular adhesion was thinner in the ESS‐treated group compared with the control (Figure 2E). Moreover, we found that ESS significantly decreased cell viability but increased ROS formation compared with the control group (both *P* <.01; Figure 2F‐H). These results showed that ESS exposure could cause HBE cell injury and decrease the transcription of IP_3_R.

### 3.4 Silencing of IP_3_R enhanced oxidative stress and inflammation damage in ESS‐treated HBE cells

**FIGURE 3:**
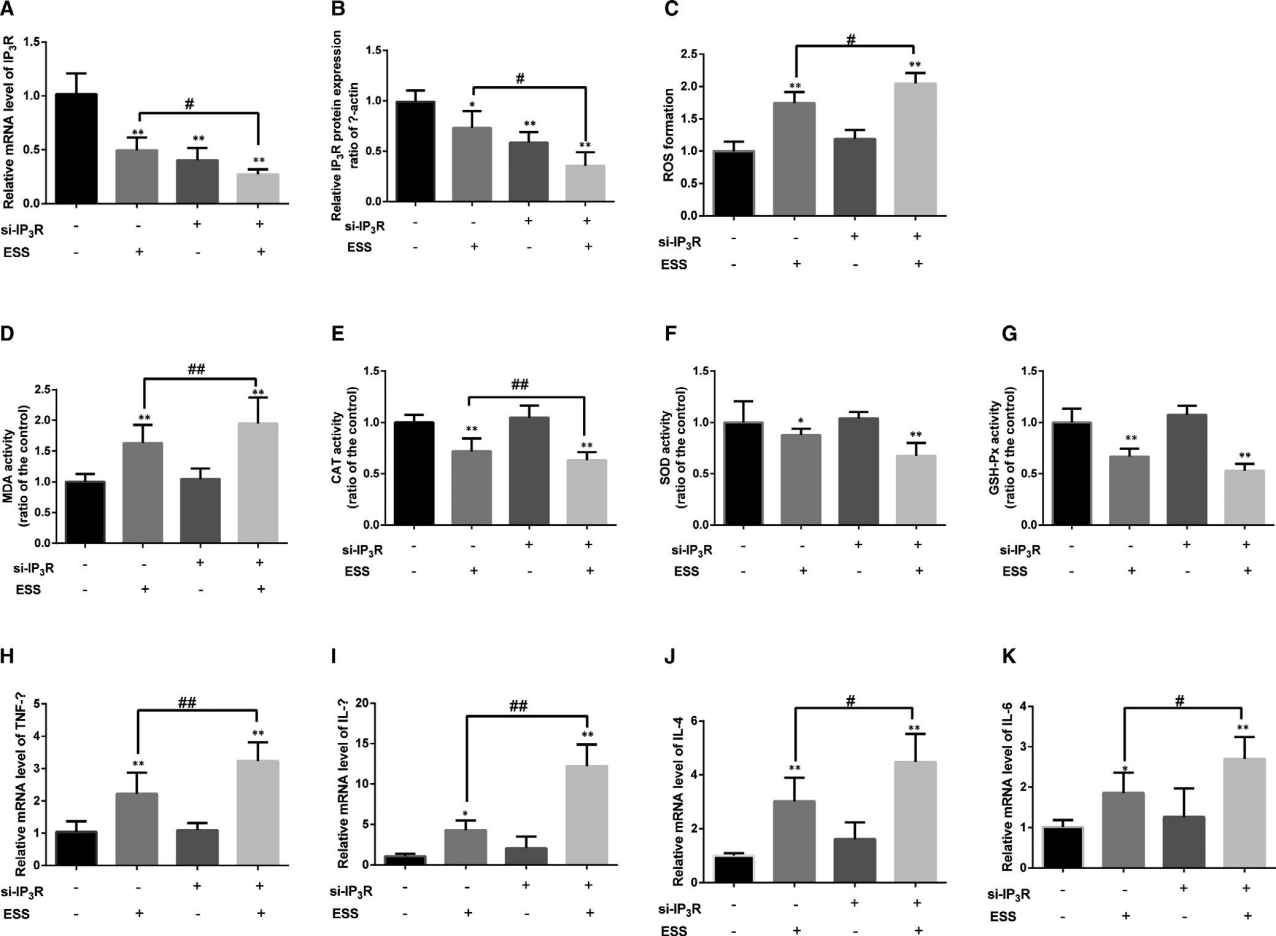
Oxidative stress level and the inflammatory response in si‐IP3R HBE cells pre‐treated with ESS. A and B, The mRNA and protein expression of IP_3_R in transfected HBE cells. CM The variation in ROS formation in si‐IP3R HBE cells after ESS treatment. D‐G, The level of MDA and the activities of CAT, SOD and GSH‐Px after exposure to ESS. H‐K, The results of RT‐qPCR analysis of TNF‐α, IL‐1β, IL‐4 and IL‐6 gene expression in si‐IP3R HBE cells pre‐treated with ESS. One single experimental band was selected to represent the results. Experiments were performed at least three times, and data are shown as the mean ± SD. *P < .05, **P < .01 compared with the control group; #*P* < .05, ##*P* < .01 compared with the single ESS‐treated HBE cells

To assess the effect of IP_3_R in ESS‐treated HBE cells, the IP_3_R was silenced by lentiviral transfection. As shown in Figure 3A‐C, both the mRNA expression and protein expression of IP_3_R were significantly decreased after ESS treatment and si‐IP_3_R transfection compared with the control group (*P* < .05 or *P* < .01). Moreover, we detected that ESS exposure significantly elevated MDA but decreased levels of CAT, SOD and GSH‐Px (*P* < .05 or *P* < .01), while si‐IP_3_R further enhanced these functions. However, there were no significant differences in SOD and GSH‐Px (Figure 3D‐G). Furthermore, as shown in Figure 3H‐K, the relative mRNA expression levels of TNF‐α, IL‐1β, IL‐4 and IL‐6 were all significantly increased by silencing of IP_3_R under ESS exposure compared with the control group (*P* < .05 or *P* < .01). These findings confirmed that silencing of IP3R could enhance oxidative stress and inflammation damage in ESS‐treated HBE cells.

### 3.5 Overexpression of IP_3_R decreased oxidative stress and inflammatory damage in ESS‐treated HBE cells

**FIGURE 4:**
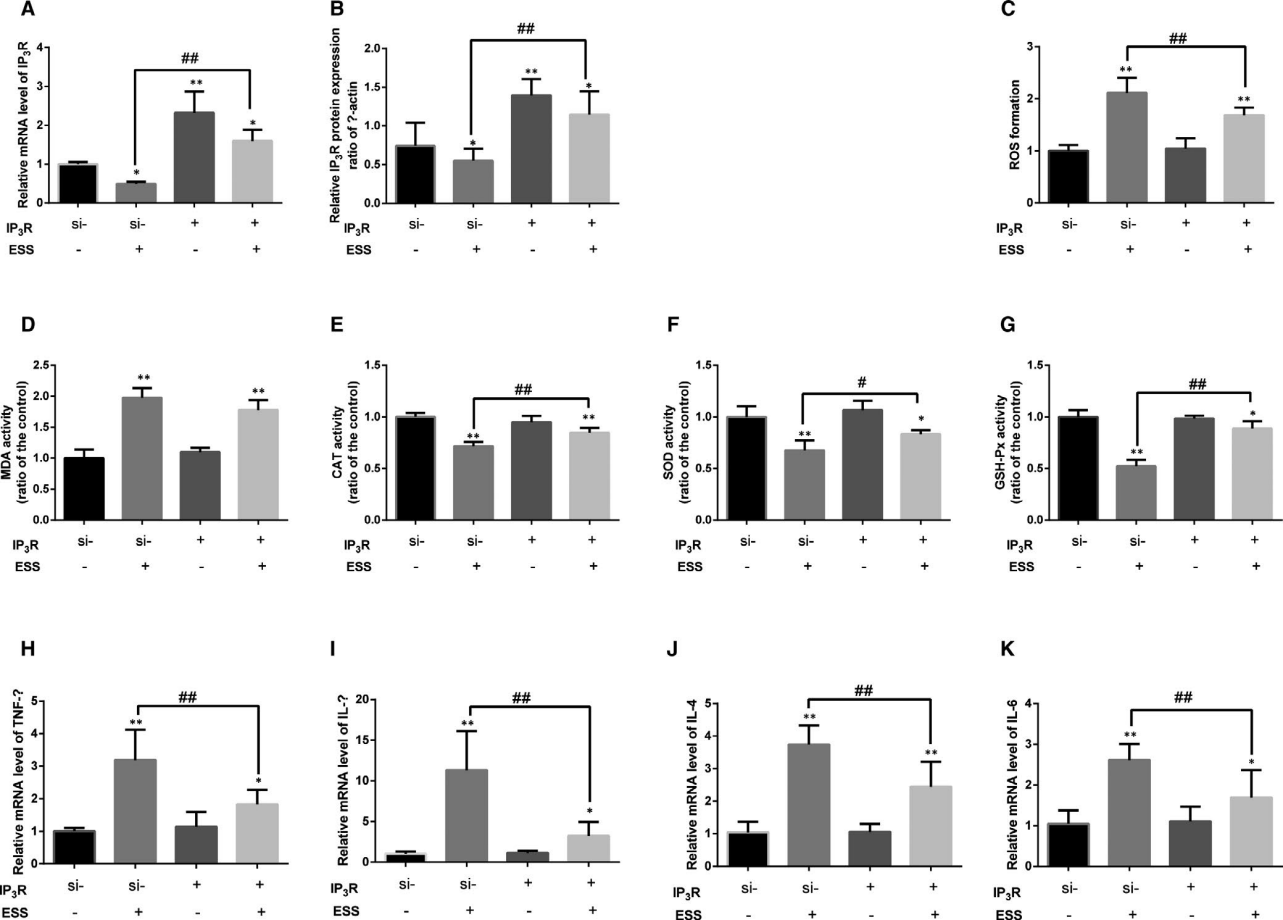
Oxidative stress level and inflammatory response in overexpressing IP_3_R HBE cells pre‐treated with ESS. A and B, The mRNA and protein expression of IP_3_R in overexpressing IP_3_R HBE cells after ESS treatment. C, The variation in ROS formation in IP3R^+^ HBE cells after ESS treatment. D‐G, The level of MDA and the activities of CAT, SOD and GSH‐Px in IP3R^+^ HBE cells after exposure to ESS. H‐K, The results of RT‐qPCR analysis of TNF‐α, IL‐1β, IL‐4 and IL‐6 gene expression in IP3R^+^ HBE cells pre‐treated with ESS. One single experimental band was selected to represent the results. Experiments were performed at least three times, and the data are shown as the mean ± SD. *P < .05, **P < .01 compared with the control group; #*P* < .05, ##*P* < .01 compared with the single ESS‐treated HBE cells

Next, we overexpressed the IP_3_R gene by lentiviral transfection in si‐IP_3_R HBE cells. The mRNA and protein expression levels of IP_3_R are shown in Figure 4A,B, respectively. The results demonstrated that both the mRNA and protein levels of IP_3_R were statistically increased in the overexpression (IP_3_R^+^) group compared with the control group (*P* < .01). The fluorescence intensity was significantly lower in the IP3R+ group than that in the control group after treatment with ESS, which indicated that ROS formation was decreased with the overexpression of IP_3_R (Figure 4C). As shown in Figure 4D, the MDA level was obviously decreased in the IP_3_R^+^ group compared with the control group (*P* < .01). Furthermore, the CAT, SOD and GSH‐Px activities were specifically increased in the IP_3_R^+^ group compared with the control group (*P* < .05 or *P* < .01; Figure 4E‐G). Additionally, the relative gene expression levels of TNF‐α, IL‐1β, IL‐4 and IL‐6 were all obviously decreased in the IP_3_R^+^ group compared with the control group (*P* < .05 or *P* < .01; Figure 4H‐K). These data implied that overexpression of IP_3_R could decrease oxidative stress and inflammatory damage in ESS‐treated HBE cells.

### 3.6 Aberrant expression of IP_3_R changed autophagy level in ESS‐treated HBE cells

**FIGURE 5:**
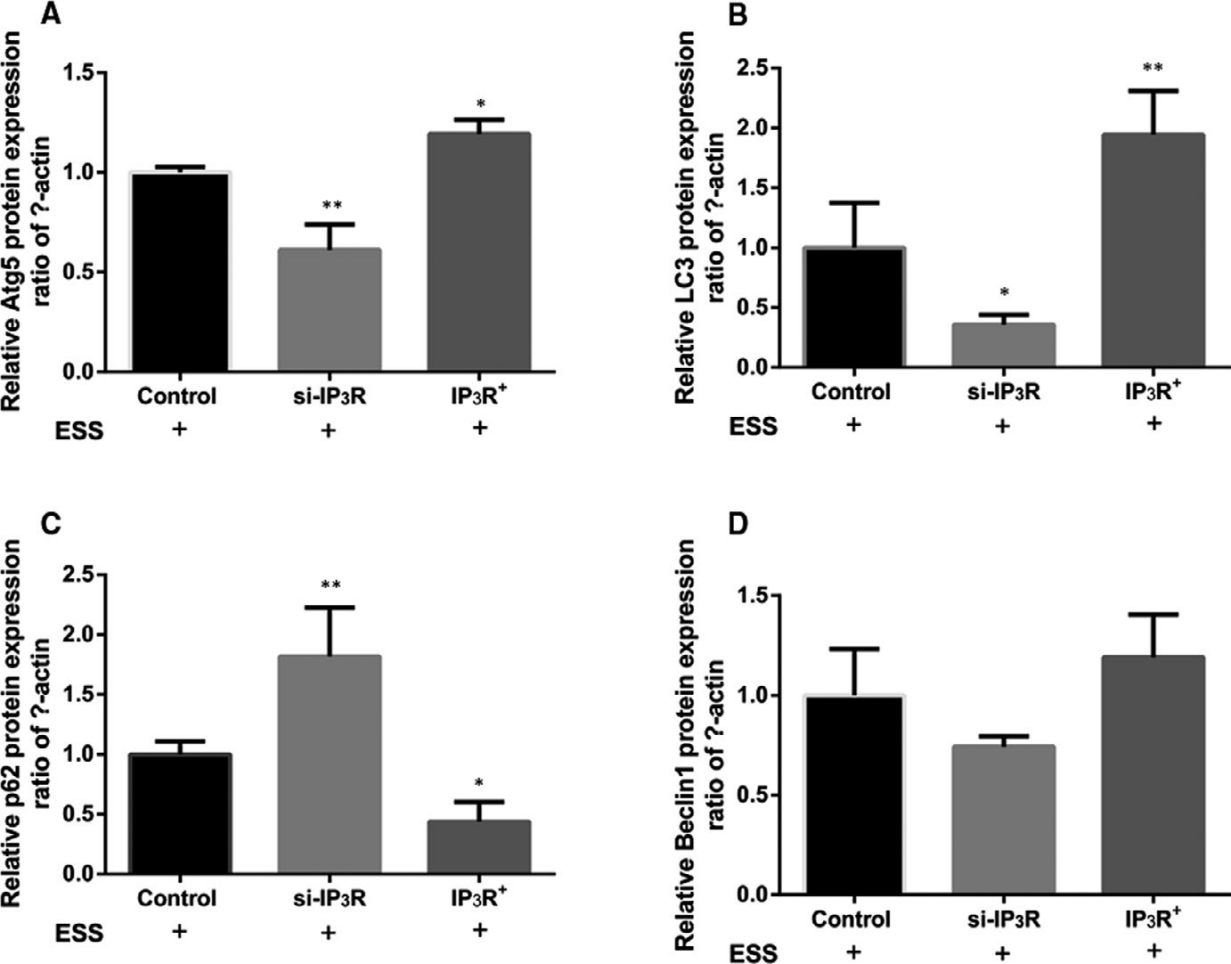
Expression of autophagy‐related proteins in ESS‐pre‐treated HBE cells. A‐D, The expression levels of the Atg5, LC3, P62 and Beclin1 proteins after overexpression or silencing of IP3R in ESS‐pre‐treated HBE cells. All experiments were performed at least three times, and similar results were obtained; the data are shown as the mean ± SD. *P < .05, **P < .01 compared with the control group

To further explore the role of IP3R in autophagy, we analysed the protein expression of Atg5, LC3, P62 and Beclin1 in ESS‐treated HBE cells. Relative expression of the Atg5 and LC3 was markedly decreased in the si‐IP_3_R group but increased in the IP_3_R^+^ group compared with the control group (*P* < .05 or *P* < .01; Figure 5A,B). In contrast, the relative protein expression of p62 was significantly increased in the si‐IP_3_R group and decreased in the IP_3_R^+^ group compared with the control group (*P* < .05 or *P* < .01; Figure 5C). Moreover, we found that the protein expression of Beclin1 was decreased in the si‐IP_3_R group and increased in the IP_3_R^+^ group; however, no significant differences were detected (Figure 5D).

### 3.7 Protective effects of IP_3_R on ESS‐treated HBE cells were through regulation of autophagy

**FIGURE 6:**
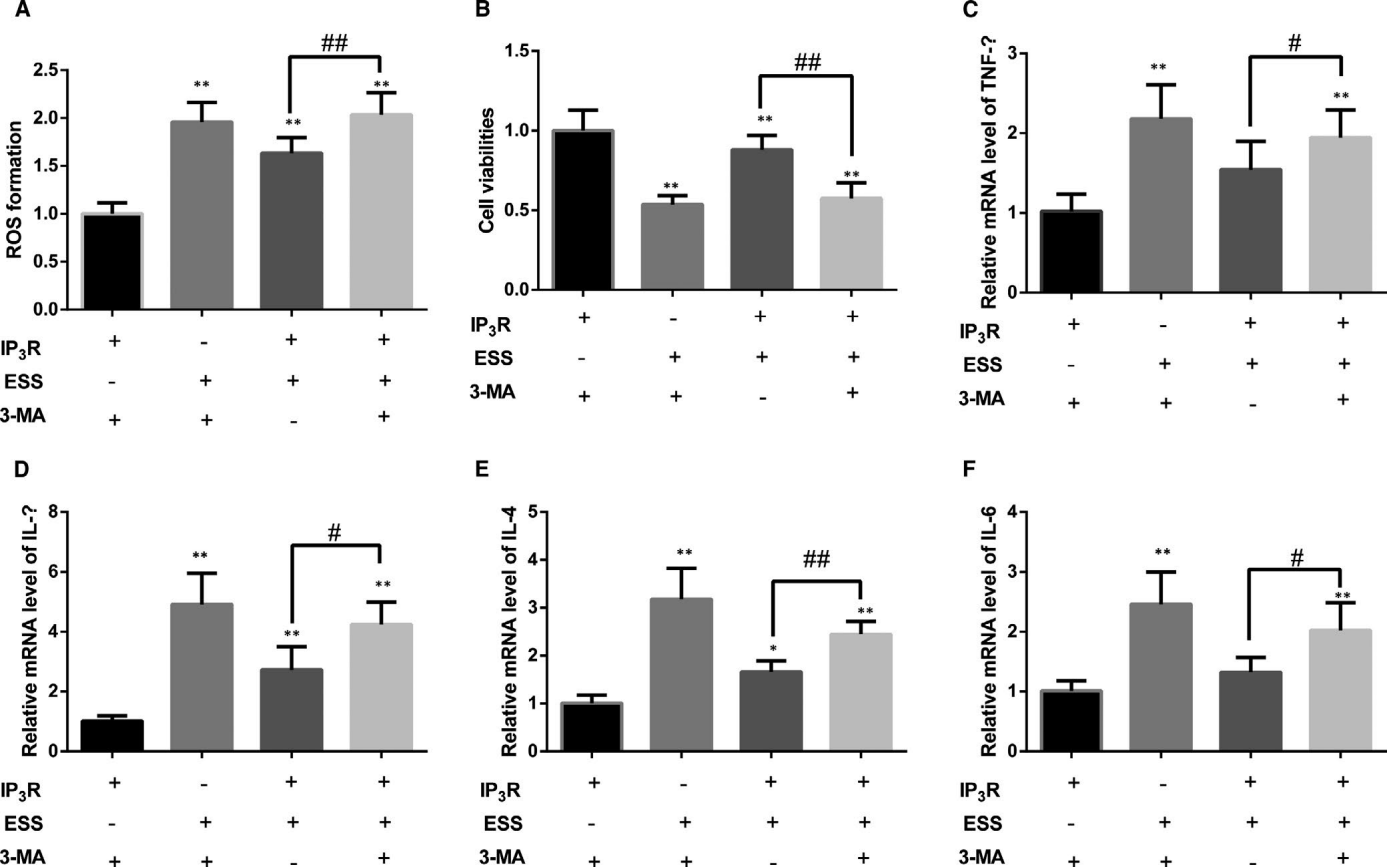
Variation in the oxidative stress level and inflammatory response in ESS‐pre‐treated IP3R^+^ HBE cells after the addition of 3‐MA. A, The variation of ROS formation in IP3R^+^ HBE cells after ESS treatment. B, The variation of cell viability after exposure to ESS. C‐F, The results of RT‐qPCR analysis of TNF‐α, IL‐1β, IL‐4 and IL‐6 gene expression in IP3R^+^ HBE cells pre‐treated with ESS. Experiments were performed at least three times, and the data are shown as the mean ± SD. *P < .05, **P < .01 compared with the no‐ESS–treated group. #*P* < .05, ##*P* < .01 compared with the no‐3‐MA–treated group

**FIGURE 7:**
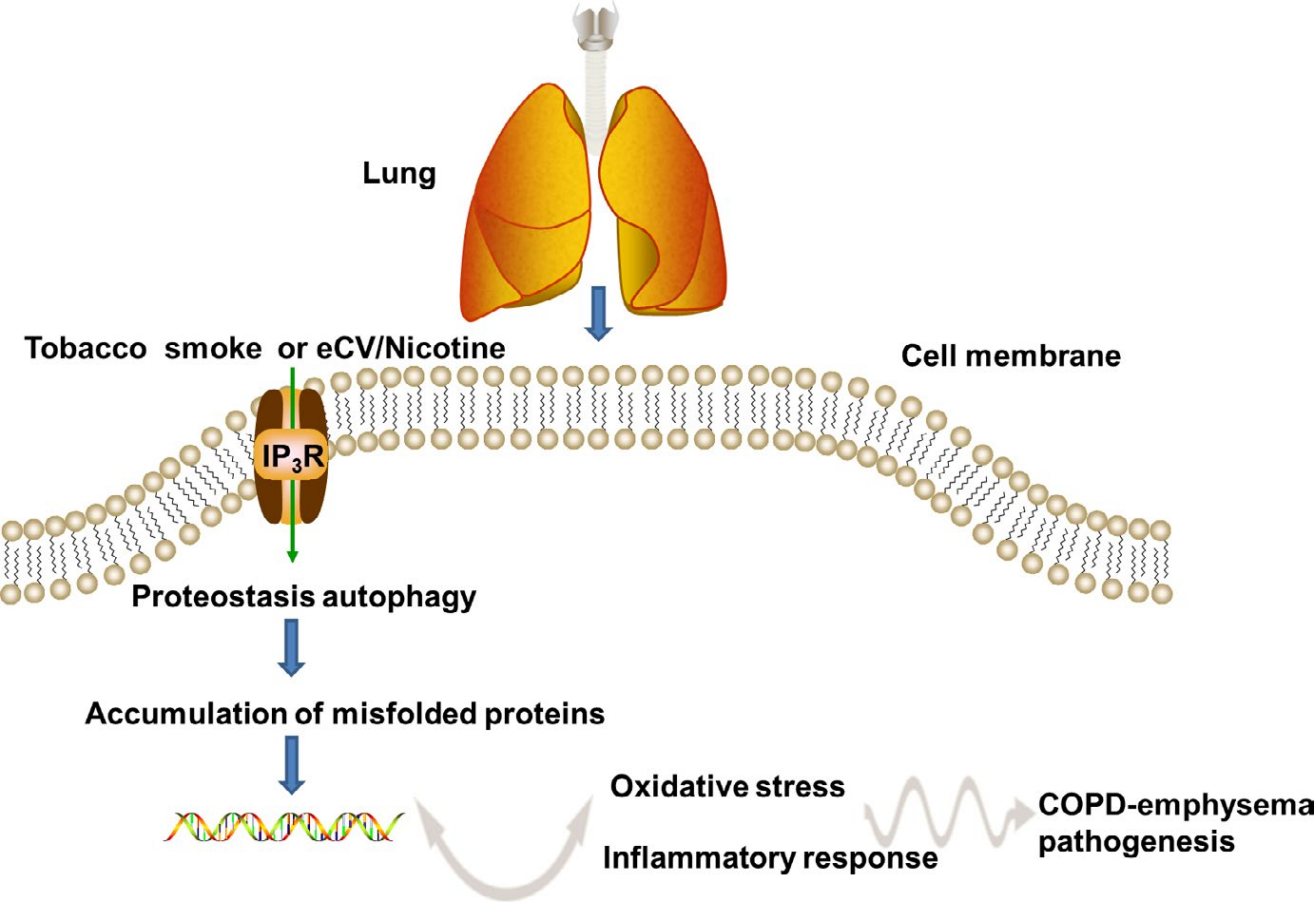
A schematic diagram of the study

To further determine whether IP_3_R had protective effects in ESS‐treated HBE cells by regulating autophagy, the cells were pre‐treated with the autophagy inhibitor 3‐MA. As shown in the figures, administration of 3‐MA significantly increased the ROS (Figure 6A), lowered cell viability (Figure 6B) and elevated the expression of inflammatory cytokines (Figure 6C‐F) under the condition of IP_3_R overexpression and ESS exposure (*P* < .05 or *P* < .01). These data suggested that the protective effects of IP3R on ESS‐treated HBE cells might be through regulation of autophagy. A schematic diagram of the study is demonstrated in Figure 7.

## 4 DISCUSSION

Numerous studies have reported the possible related mechanisms of COPD. The pathogenic mechanisms of tobacco smoke were previously reported to be closely associated with the inflammatory response, oxidative damage and autophagy in the airway.^4^, ^47^, ^48^ However, the most critical gene and protein in tobacco smoke–induced COPD remain poorly understood. Therefore, this study aimed to investigate variations in this hub gene and protein in tobacco smoke–induced COPD and further explore the role of this key target.

In recent years, bioinformatics analysis has been widely used to determine increasingly reliable and crucial biomarkers of diseases.^49^, ^50^, ^51^ We analysed gene expression data from GEO data sets, to identify the DEGs common to tobacco smoke‐induced COPD patient and normal smoker samples, both compared with that in volunteer non‐smoker controls. The use of GEO2R and Venn software revealed 30 DEGs common to tobacco smoke–induced COPD patient and normal smoker samples. Fundamentally, the samples from the normal smoker volunteers still belonged to the normal group, and it is conceivable that there are not a large number of DEGs common to the two groups. In patients with COPD, these common DEGs were considered smoke‐sensitive genes. Subsequently, we further enriched gene‐ and pathway‐related information via GO and KEGG analyses. Based on the results of BP, MF, and CC and KEGG pathway analyses, we screened several crucial genes (*IP_3_R*, *PLA2G2A* and *NOTCH3*). Previous studies have proposed that the autophagy acts as a novel strategy to control COPD‐emphysema pathogenesis.^31^, ^52^, ^53^ In addition, oxidative stress and inflammation, which are closely related to the progresses of COPD, have been demonstrated their roles in promoting autophagy‐flux impairment. These three factors could work together to further disequilibrate the clearance of incorrectly folded proteins and bacterial/viral pathogens, leading to cell death.^54^ Furthermore, it has been revealed that IP_3_R is associated with autophagy.^55^ Inhibition of IP_3_R with a particular antagonist, such as xestospongin B, or knockdown of IP_3_R with small interfering RNAs (siRNAs) is a strong stimulus of autophagy.^56^ In consideration of the association between autophagy and oxidative stress and inflammation and association between autophagy and IP_3_R, we speculated that IP_3_R‐mediated autophagy might be participated in the occurrence and development of COPD by regulation of oxidative stress and inflammation. To confirm our speculation, we firstly determined the expression of IP_3_R in patients with COPD by analysing *IP_3_R* gene expression in the GES103174 data set and found that the mRNA expression level of *IP_3_R* was decreased in tobacco smoke–induced COPD patients compared with the non‐smoker controls. Similar results were observed when peripheral blood samples from COPD patients (who had smoked for more than 15 years) and volunteer non‐smokers were compared. In addition, we also observed that both the mRNA expression and protein expression of IP_3_R were markedly decreased in ESS‐treated HBE cells compared with control cells.

To further explore the functional role of IP_3_R in COPD, the effects of IP_3_R silencing and overexpressing on oxidative stress and the inflammatory response were observed in ESS‐treated HBE cells. As demonstrated in our data, silencing of IP_3_R could significantly elevate the ROS formation and activities of MDA and CAT, but decrease the activities of SOD and GSH‐Px, whereas exposure to ESS could further promote the silencing of IP_3_R‐induced oxidative stress injury. However, we found that overexpressing of IP_3_R reversed the above effects. This information indicated that IP_3_R had a protective function in ESS‐induced HBE cell injury through decreasing oxidative stress. These results might be associated with variation in Ca^2+^ release induced by IP_3_R, which can influence the generation of mitochondrial ROS.^57^ Our results were similar with previous studies, in which the authors have confirmed that the redox sensitivity of IP_3_R augments oxidative injury by producing positive feedback to accelerate the generation of mitochondrial ROS and to escalate ER Ca^2+^ release and mitochondrial Ca^2+^ loading.^58^, ^59^ However, the exact underlying mechanism(s) of the functions of IP_3_R on oxidative stress need to be further explored. Furthermore, in this study, we showed that the loss of IP_3_R increased the level of inflammation‐related cytokines, including TNF‐α, IL‐1β, IL‐4 and IL‐6, compared with those in the control group. Nevertheless, this consequence was reversed in ESS‐treated IP_3_R^+^ HBE cells. These results indicated that IP_3_R might negatively regulate the ESS‐induced inflammatory response in HBE cells. Interestingly, the effects of IP_3_R on inflammatory response were in line with a previous study demonstrating IP_3_R inhibitor 2APB attenuated inflammation in CD36‐overexpressing preadipocytes.^60^

Autophagy is a widespread activity in the inflammatory response of COPD,^29^, ^30^ and autophagy was shown to be induced by the relatively excessive accumulation of ROS.^61^ In the present study, we observed variations in a series of autophagy‐related proteins in ESS‐treated si‐IP_3_R HBE cells and IP_3_R^+^ HBE cells compared with control cells. However, the regulatory mechanism of IP_3_R on autophagy is still not obvious. A previous research has confirmed that IP_3_ binds to IP_3_R on the ER and induces the release of intracellular Ca^2+^ stores. The high level of intracellular Ca^2+^ activates calmodulin, thereby blocking autophagy.^62^ In addition, it has reported that endoplasmic reticulum stress (ERS)–induced IP_3_R can lead to the occurrence of autophagy through different signal transduction pathways, but the mechanism of ERS that can cause autophagy through IP_3_R remains not obvious.^63^ Therefore, we suspected that the effects of IP_3_R on ESS‐treated HBE cells may be also due to the involvement of autophagy. To further verify our speculation, we investigated autophagy‐related proteins. An increase in Beclin1, LC3 and Atg5 indicates active cell autophagy.^64^ Furthermore, p62, an autophagy adaptor protein, could mediate Keap1 inactivation and induce the accumulation of Nrf2, finally causing inflammation.^65^ In the present study, as shown by variations in autophagy‐related protein expression, the autophagy level was decreased in si‐IP_3_R HBE cells but increased in IP_3_R^+^ HBE cells compared with control cells after treatment with ESS. To further investigate whether the protective function of IP_3_R in HBE cells was achieved through autophagy, autophagy was inhibited in ESS‐treated IP_3_R^+^ HBE cells with an inhibitor. As shown in our data, the protective function of IP_3_R was decreased in the inhibitor group. Based on these results, we suspected that IP_3_R might play a protective role in ESS‐induced inflammation and oxidative stress in HBE cells and that the function of IP_3_R might be achieved associated with the autophagy‐related proteins. However, a more detailed upstream mechanism and the molecular targets associated with the regulation of IP_3_R remain not obvious. In addition, functional in vivo animal model, such as knockout animal experiments, should be performed to verify the antioxidant and anti‐inflammatory properties of IP_3_R, as well as the regulatory mechanism of IP_3_R on autophagy.

In summary, the study demonstrated that IP_3_R had protective effects in ESS‐treated HBE cells and suggests IP_3_R as a potential target to prevent tobacco smoke–induced COPD.

## CONFLICT OF INTEREST

The authors declare that they have no competing interests.

## AUTHOR CONTRIBUTIONS

**Qiang Zhang:** Data curation (lead); Investigation (equal); Methodology (equal); Supervision (equal); Visualization (equal); Writing‐original draft (equal). **Wei Li:** Investigation (equal); Software (equal). **Nahemuguli Ayidaerhan:** Data curation (equal); Investigation (equal); Resources (equal); Software (equal). **Wuxin Han:** Formal analysis (equal); Project administration (equal); Visualization (equal). **Yingying Chen:** Data curation (equal); Methodology (equal); Software (equal). **Wei Song:** Investigation (equal); Methodology (equal); Validation (equal). **Yuanyi Yue:** Conceptualization (lead); Investigation (equal); Supervision (equal); Writing‐original draft (equal); Writing‐review & editing (equal).

## Supporting information

Figure S1

## Data Availability

The data sets used and/or analysed during the current study are available from the corresponding author on reasonable request.

## References

[1] Brandsma CA , Van den Berge M , Hackett TL , Brusselle G , Timens W . Recent advances in chronic obstructive pulmonary disease pathogenesis: from disease mechanisms to precision medicine. J Pathol. 2020;250:624‐635. 31691283 10.1002/path.5364 PMC7216938

[2] Lundbäck B , Lindberg A , Lindström M , et al. Not 15 but 50% of smokers develop COPD? – report from the Obstructive Lung Disease in Northern Sweden Studies. Respir Med. 2003;97:115‐122. 12587960 10.1053/rmed.2003.1446

[3] Salvi S . Tobacco smoking and environmental risk factors for chronic obstructive pulmonary disease. Clin Chest Med. 2014;35:17‐27. 24507834 10.1016/j.ccm.2013.09.011

[4] Zhou Z , Chen P , Peng H . Are healthy smokers really healthy. Tob Induc Dis. 2016;14:35. 27891067 10.1186/s12971-016-0101-z PMC5111288

[5] Zong D , Liu X , Li J , Ouyang R , Chen P . The role of cigarette smoke‐induced epigenetic alterations in inflammation. Epigenetics Chromatin. 2019;12:65. 31711545 10.1186/s13072-019-0311-8 PMC6844059

[6] Karimi L , Lahousse L , Ghanbari M , et al. β(2)‐Adrenergic receptor (ADRB2) gene polymorphisms and risk of COPD exacerbations: the Rotterdam Study. J Clin Med. 2019;8:1835. 10.3390/jcm8111835 PMC6912270 31683975

[7] Ortega VE , Li X , O'Neal WK , et al. The effects of rare SERPINA1 variants on lung function and emphysema in SPIROMICS. Am J Respir Crit Care Med. 2020;201:540‐554. 31661293 10.1164/rccm.201904-0769OC PMC7047460

[8] Thillaiappan NB , Chakraborty P , Hasan G , Taylor CW . IP(3) receptors and Ca(2+) entry. Biochim Biophys Acta. 2019;1866:1092‐1100. 10.1016/j.bbamcr.2018.11.007 30448464

[9] Prole DL , Taylor CW . Structure and function of IP(3) receptors. Cold Spring Harbor Perspect Biol. 2019;11 :a035063. 10.1101/cshperspect.a035063 PMC6442203 30745293

[10] Joseph SK , Young MP , Alzayady K , Yule DI . Redox regulation of type‐I inositol trisphosphate receptors in intact mammalian cells. J Biol Chem. 2018;293:17464‐17476. 30228182 10.1074/jbc.RA118.005624 PMC6231128

[11] Wagner LE 2nd , Yule DI . Differential regulation of the InsP₃ receptor type‐1 and ‐2 single channel properties by InsP₃, Ca^2^⁺ and ATP. J Physiol. 2012;590:3245‐3259. 22547632 10.1113/jphysiol.2012.228320 PMC3459040

[12] Taylor CW . Regulation of IP(3) receptors by cyclic AMP. Cell Calcium. 2017;63:48‐52. 27836216 10.1016/j.ceca.2016.10.005 PMC5471599

[13] Cárdenas C , Müller M , McNeal A , et al. Selective vulnerability of cancer cells by inhibition of Ca(2+) transfer from endoplasmic reticulum to mitochondria. Cell Rep. 2016;15:219‐220. 27050774 10.1016/j.celrep.2016.03.045

[14] La Rovere RM , Roest G , Bultynck G , Parys JB . Intracellular Ca(2+) signaling and Ca(2+) microdomains in the control of cell survival, apoptosis and autophagy. Cell Calcium. 2016;60:74‐87. 27157108 10.1016/j.ceca.2016.04.005

[15] Xu H , Ren D . Lysosomal physiology. Annu Rev Physiol. 2015;77:57‐80. 25668017 10.1146/annurev-physiol-021014-071649 PMC4524569

[16] Egorova PA , Bezprozvanny IB . Inositol 1,4,5‐trisphosphate receptors and neurodegenerative disorders. FEBS J. 2018;285:3547‐3565. 29253316 10.1111/febs.14366

[17] Hisatsune C , Mikoshiba K . IP(3) receptor mutations and brain diseases in human and rodents. J Neurochem. 2017;141:790‐807. 28211945 10.1111/jnc.13991

[18] Messai Y , Noman MZ , Hasmim M , et al. ITPR1 protects renal cancer cells against natural killer cells by inducing autophagy. Cancer Res. 2014;74:6820‐6832. 25297632 10.1158/0008-5472.CAN-14-0303

[19] Lin BH , Tsai MH , Lii CK , Wang TS . IP3 and calcium signaling involved in the reorganization of the actin cytoskeleton and cell rounding induced by cigarette smoke extract in human endothelial cells. Environ Toxicol. 2016;31:1293‐1306. 25758670 10.1002/tox.22133

[20] Montuschi P , Collins JV , Ciabattoni G , et al. Exhaled 8‐isoprostane as an in vivo biomarker of lung oxidative stress in patients with COPD and healthy smokers. Am J Respir Crit Care Med. 2000;162:1175‐1177. 10988150 10.1164/ajrccm.162.3.2001063

[21] Kirkham PA , Barnes PJ . Oxidative stress in COPD. Chest. 2013;144:266‐273. 23880677 10.1378/chest.12-2664

[22] Zang LY , Stone K , Pryor WA . Detection of free radicals in aqueous extracts of cigarette tar by electron spin resonance. Free Radic Biol Med. 1995;19:161‐167. 7649487 10.1016/0891-5849(94)00236-d

[23] Halliwell B . Antioxidants in human health and disease. Annu Rev Nutr. 1996;16:33‐50. 8839918 10.1146/annurev.nu.16.070196.000341

[24] Malhotra D , Thimmulappa R , Navas‐Acien A , et al. Decline in NRF2‐regulated antioxidants in chronic obstructive pulmonary disease lungs due to loss of its positive regulator, DJ‐1. Am J Respir Crit Care Med. 2008;178:592‐604. 18556627 10.1164/rccm.200803-380OC PMC2542433

[25] Barnes PJ . The cytokine network in asthma and chronic obstructive pulmonary disease. J Clin Investig. 2008;118:3546‐3556. 18982161 10.1172/JCI36130 PMC2575722

[26] Aaron SD , Angel JB , Lunau M , et al. Granulocyte inflammatory markers and airway infection during acute exacerbation of chronic obstructive pulmonary disease. Am J Respir Crit Care Med. 2001;163:349‐355. 11179105 10.1164/ajrccm.163.2.2003122

[27] Fischer BM , Voynow JA , Ghio AJ . COPD: balancing oxidants and antioxidants. Int J Chron Obstruct Pulmon Dis. 2015;10:261‐276. 25673984 10.2147/COPD.S42414 PMC4321570

[28] Mizushima N , Komatsu M . Autophagy: renovation of cells and tissues. Cell. 2011;147:728‐741. 22078875 10.1016/j.cell.2011.10.026

[29] Dickinson JD , Alevy Y , Malvin NP , et al. IL13 activates autophagy to regulate secretion in airway epithelial cells. Autophagy. 2016;12:397‐409. 26062017 10.1080/15548627.2015.1056967 PMC4835964

[30] Abdel Fattah E , Bhattacharya A , Herron A , Safdar Z , Eissa NT . Critical role for IL‐18 in spontaneous lung inflammation caused by autophagy deficiency. J Immunol. 2015;194:5407‐5416. 25888640 10.4049/jimmunol.1402277 PMC4433854

[31] Bodas M , Vij N . Augmenting autophagy for prognosis based intervention of COPD‐pathophysiology. Respir Res. 2017;18:83. 28472967 10.1186/s12931-017-0560-7 PMC5418861

[32] Vij N , Chandramani‐Shivalingappa P , Van Westphal C , Hole R , Bodas M . Cigarette smoke‐induced autophagy impairment accelerates lung aging, COPD‐emphysema exacerbations and pathogenesis. Am J Physiol Cell Physiol. 2018;314:C73‐c87. 27413169 10.1152/ajpcell.00110.2016 PMC5866380

[33] Bodas M , Patel N , Silverberg D , Walworth K , Vij N . Master autophagy regulator transcription factor EB regulates cigarette smoke‐induced autophagy impairment and chronic obstructive pulmonary disease‐emphysema pathogenesis. Antioxid Redox Signal. 2017;27:150‐167. 27835930 10.1089/ars.2016.6842 PMC5510670

[34] Bodas M , Van Westphal C , Carpenter‐Thompson R , Mohanty DK , Vij N . Nicotine exposure induces bronchial epithelial cell apoptosis and senescence via ROS mediated autophagy‐impairment. Free Radic Biol Med. 2016;97:441‐453. 27394171 10.1016/j.freeradbiomed.2016.06.017

[35] Bodas M , Min T , Vij N . Lactosylceramide‐accumulation in lipid‐rafts mediate aberrant‐autophagy, inflammation and apoptosis in cigarette smoke induced emphysema. Apoptosis. 2015;20:725–739. 25638276 10.1007/s10495-015-1098-0

[36] Tran I , Ji C , Ni I , Min T , Tang D , Vij N . Role of cigarette smoke‐induced aggresome formation in chronic obstructive pulmonary disease‐emphysema pathogenesis. Am J Respir Cell Mol Biol. 2015;53:159‐173. 25490051 10.1165/rcmb.2014-0107OC PMC5455694

[37] Yamada Y , Tomaru U , Ishizu A , et al. Decreased proteasomal function accelerates cigarette smoke‐induced pulmonary emphysema in mice . Lab Invest. 2015;95:625‐634. 25915723 10.1038/labinvest.2015.43

[38] Decuypere JP , Paudel RC , Parys J , Bultynck G . Intracellular Ca(2+) signaling: a novel player in the canonical mTOR‐controlled autophagy pathway. Commun Integr Biol. 2013;6:e25429. 24265855 10.4161/cib.25429 PMC3829968

[39] Decuypere JP , Welkenhuyzen K , Luyten T , et al. Ins(1,4,5)P3 receptor‐mediated Ca2+ signaling and autophagy induction are interrelated. Autophagy. 2011;7:1472‐1489. 22082873 10.4161/auto.7.12.17909 PMC3327615

[40] Decuypere JP , Kindt D , Luyten T , et al. mTOR‐controlled autophagy requires intracellular Ca(2+) signaling. PLoS One. 2013;8:e61020. 23565295 10.1371/journal.pone.0061020 PMC3614970

[41] Benito M , Parker J , Du Q , et al. Adjustment of systematic microarray data biases. Bioinformatics. 2004;20:105‐114. 14693816 10.1093/bioinformatics/btg385

[42] da Huang W , Sherman BT , Lempicki RA . Bioinformatics enrichment tools: paths toward the comprehensive functional analysis of large gene lists. Nucleic Acids Res. 2009;37:1‐13. 19033363 10.1093/nar/gkn923 PMC2615629

[43] da Huang W , Sherman BT , Lempicki RA . Systematic and integrative analysis of large gene lists using DAVID bioinformatics resources. Nat Protoc. 2009;4:44‐57. 19131956 10.1038/nprot.2008.211

[44] Yang SR , Wright J , Bauter M , Seweryniak K , Kode A , Rahman I . Sirtuin regulates cigarette smoke‐induced proinflammatory mediator release via RelA/p65 NF‐kappaB in macrophages in vitro and in rat lungs in vivo: implications for chronic inflammation and aging. Am J Physiol Lung Cell Mol Physiol. 2007;292:L567‐L576. 17041012 10.1152/ajplung.00308.2006

[45] Sun X , Feng X , Zheng D , et al. Ergosterol attenuates cigarette smoke extract‐induced COPD by modulating inflammation, oxidative stress and apoptosis in vitro and in vivo. Clin Sci. 2019;133:1523‐1536. 10.1042/CS20190331 31270147

[46] Cheng WW , Lin ZQ , Wei BF , et al. Single‐walled carbon nanotube induction of rat aortic endothelial cell apoptosis: reactive oxygen species are involved in the mitochondrial pathway. Int J Biochem Cell Biol. 2011;43:564‐572. 21172451 10.1016/j.biocel.2010.12.013

[47] Fujii S , Hara H , Araya J , et al. Insufficient autophagy promotes bronchial epithelial cell senescence in chronic obstructive pulmonary disease. Oncoimmunology. 2012;1:630‐641. 22934255 10.4161/onci.20297 PMC3429567

[48] Tan WSD , Shen HM , Wong WSF . Dysregulated autophagy in COPD: a pathogenic process to be deciphered. Pharmacol Res. 2019;144:1‐7. 30953685 10.1016/j.phrs.2019.04.005

[49] Guo T , Ma H , Zhou Y . Bioinformatics analysis of microarray data to identify the candidate biomarkers of lung adenocarcinoma. PeerJ. 2019;7:e7313. 31333911 10.7717/peerj.7313 PMC6626531

[50] Chen N , Hao C , Peng X , et al. Roxadustat for anemia in patients with kidney disease not receiving dialysis. N Engl J Med. 2019;381:1001‐1010. 31340089 10.1056/NEJMoa1813599

[51] Zhang Q , Song W , Ayidaerhan N , He Z . PTPLAD2 and USP49 involved in the pathogenesis of smoke‐induced COPD by Integrative Bioinformatics Analysis. Int J Chron Obstruct Pulmon Dis. 2020;15:2515‐2526. 33116468 10.2147/COPD.S250576 PMC7571584

[52] Mizumura K , Maruoka S , Shimizu T , Gon Y . Autophagy, selective autophagy, and necroptosis in COPD. Int J Chron Obstruct Pulmon Dis. 2018;13:3165‐3172. 30349225 10.2147/COPD.S175830 PMC6186766

[53] Kuwano K , Araya J , Hara H , et al. Cellular senescence and autophagy in the pathogenesis of chronic obstructive pulmonary disease (COPD) and idiopathic pulmonary fibrosis (IPF). Respir Invest. 2016;54:397‐406. 10.1016/j.resinv.2016.03.010 27886850

[54] Shivalingappa PC , Hole R , Westphal CV , Vij N . Airway exposure to E‐cigarette vapors impairs autophagy and induces aggresome formation. Antioxid Redox Signal. 2016;24:186‐204. 26377848 10.1089/ars.2015.6367 PMC4744882

[55] Criollo A , Vicencio JM , Tasdemir E , Maiuri MC , Lavandero S , Kroemer G . The inositol trisphosphate receptor in the control of autophagy. Autophagy. 2007;3:350‐353. 17404493 10.4161/auto.4077

[56] Criollo A , Maiuri MC , Tasdemir E , et al. Regulation of autophagy by the inositol trisphosphate receptor. Cell Death Differ. 2007;14:1029‐1039. 17256008 10.1038/sj.cdd.4402099

[57] Görlach A , Bertram K , Hudecova S , Krizanova O . Calcium and ROS: a mutual interplay. Redox Biol. 2015;6:260‐271. 26296072 10.1016/j.redox.2015.08.010 PMC4556774

[58] Luciani DS , Gwiazda KS , Yang TL , et al. Roles of IP3R and RyR Ca2+ channels in endoplasmic reticulum stress and beta‐cell death. Diabetes. 2009;58:422‐432. 19033399 10.2337/db07-1762 PMC2628616

[59] Lock JT , Sinkins WG , Schilling WP . Protein S‐glutathionylation enhances Ca2+‐induced Ca2+ release via the IP3 receptor in cultured aortic endothelial cells. J Physiol. 2012;590:3431‐3447. 22855054 10.1113/jphysiol.2012.230656 PMC3547261

[60] Luo X , Li Y , Yang P , et al. Obesity induces preadipocyte CD36 expression promoting inflammation via the disruption of lysosomal calcium homeostasis and lysosome function. EBioMedicine. 2020;56:102797. 32516742 10.1016/j.ebiom.2020.102797 PMC7281849

[61] Li L , Tan J , Miao Y , Lei P , Zhang Q . ROS and autophagy: interactions and molecular regulatory mechanisms. Cell Mol Neurobiol. 2015;35:615‐621. 25722131 10.1007/s10571-015-0166-x PMC11486209

[62] Harris H , Rubinsztein DC . Control of autophagy as a therapy for neurodegenerative disease. Nat Rev Neurol. 2011;8:108‐117. 22187000 10.1038/nrneurol.2011.200

[63] Qi Z , Chen L . Endoplasmic reticulum stress and autophagy. Adv Exp Med Biol. 2019;1206:167‐177. 31776985 10.1007/978-981-15-0602-4_8

[64] Chen ZH , Wu YF , Wang PL , et al. Autophagy is essential for ultrafine particle‐induced inflammation and mucus hyperproduction in airway epithelium. Autophagy. 2016;12:297‐311. 26671423 10.1080/15548627.2015.1124224 PMC5044763

[65] Komatsu M , Kurokawa H , Waguri S , et al. The selective autophagy substrate p62 activates the stress responsive transcription factor Nrf2 through inactivation of Keap1. Nat Cell Biol. 2010;12:213‐223. 20173742 10.1038/ncb2021

