# Supplementary figures and images for: IP3R attenuates oxidative stress and inflammation damage in smoking‐induced COPD by promoting autophagy
Source: J Cell Mol Med. 2021 May 31;25(13):6174–87. doi: 10.1111/jcmm.16546 (PMC8256356; doi:10.1111/jcmm.16546)

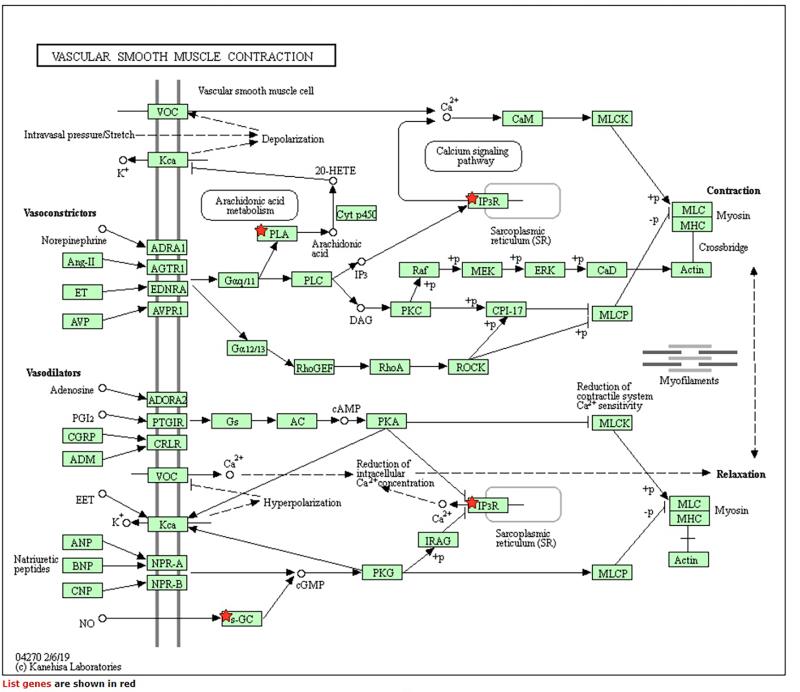

Supplement: Supplementary file 1 — Figure S1 [file JCMM-25-6174-s001.jpg]
